# Clinical Effectiveness of Training for Awareness Resilience and Action Online Compared to Standard Treatment for Adolescents and Young Adults With Depression: Study Protocol and Analysis Plan for a Pragmatic, Multi-Center Randomized Controlled Superiority Trial

**DOI:** 10.3389/fpsyt.2021.674583

**Published:** 2021-10-11

**Authors:** Erik Ekbäck, Gabriel Granåsen, Rachel Svärling, Ida Blomqvist, Eva Henje

**Affiliations:** ^1^Department of Clinical Science, Umeå University, Umeå, Sweden; ^2^Department of Public Health and Clinical Medicine, Umeå University, Umeå, Sweden

**Keywords:** adolescent depression, young adults, randomization, yoga, psychotherapy, antidepressant drugs, biomarkers

## Abstract

Depression in adolescents and young adults is an increasing global health concern. Available treatments are not sufficiently effective and relapse rates remain high. The novel group-treatment program “Training for Awareness, Resilience and Action” (TARA) targets specific mechanisms based on neuroscientific findings in adolescent depression. TARA is framed within the National Institute of Mental Health's Research Domain Criteria and has documented feasibility and preliminary efficacy in the treatment of adolescent depression. Since neurodevelopment continues well into the mid-twenties, age-adapted treatments are warranted also for young adults. Patients 15–22 years old, with either major depressive disorder (MDD) or persistent depressive disorder (PDD) according to the DSM-IV/5 or a rating >40 on the clinician rating scale Children's Depression Rating Scale—Revised (CDRS-R), will be recruited from specialized Child and Adolescent Psychiatry and local Youth-Clinics and randomized to either TARA or standard treatment, including but not limited to antidepressant medication and/or psychotherapy. Outcome measures will be obtained before randomization (T_0_), after 3 months of treatment (T_1_) and at 6-months- (T_2_) and 24-months- (T_3_) follow-up. Additionally, dose-response measures will be obtained weekly in the TARA-arm and measures for mediation-analysis will be obtained halfway through treatment (T_0.5_). Primary outcome measure is Reynolds Adolescent Depression Scale (RADS-2) score at T_1_. Secondary outcome measures include RADS-2 score at T_2_, Multidimensional Anxiety Scale for Children at T_1_ and T_2_, and CDRS-R at T_1_. Additional outcome measures include self-report measures of depression-associated symptoms, systemic bio-indicators of depression from blood and hair, heartrate variability, brain magnetic resonance imaging, as well as three-axial accelerometry for sleep-objectivization. Qualitative data will be gathered to reach a more comprehensive understanding of the factors affecting adolescents and young adults with depression and the extent to which the different treatments address these factors. In summary, this article describes the design, methods and statistical analysis plan for pragmatically evaluating the clinical effectiveness of TARA. This will be the first RCT to examine the effects of TARA compared to standard treatment for adolescents and young adults with MDD or PDD. We argue that this study will extend the current knowledgebase regarding the treatment of depression.

**NCT Registration:** identifier [NCT04747340].

## Introduction

Major depressive disorder (MDD) is a global health concern and is currently the single leading cause of disability worldwide (1). MDD is predicted by the World Health Organization to be the single largest contributor to the overall global burden of disease, measured in disability adjusted life years, by 2030 (2). Adolescence is a critical and vulnerable developmental period, in which a sharp increase in the onset of MDD has been observed, with a current point prevalence of 11% among 13–18-year-olds in the U.S. (3). Recurrent depressive episodes persisting into adulthood are common and early onset MDD predicts a threefold increase in the risk of developing adult depression (4). Traditional treatment methods for adolescent depression such as Selective serotonin reuptake inhibitors (SSRIs) and psychological treatments such as cognitive behavioral therapy (CBT) have not been sufficiently effective to slow down the increasing prevalence of depressive disorders and increased global treatment resources have been warranted (5).

According to repeated Cochrane reviews there is limited evidence upon which to draw conclusions about the relative effectiveness of psychological interventions, antidepressant medication or a combination of these interventions for adolescent depression (6). The authors conclude that “on the basis of currently available evidence, the effectiveness of these interventions for treating depressive disorders in children and adolescents cannot be established” (6). Findings from several large meta-analyses conducted since then have been consistent with this conclusion (7–11).

The rationale for using SSRIs and CBT in adolescent MDD is mainly based on studies of adults even though it is well-known that the adolescent brain and its depressive psychopathology is fundamentally different from that of adults (12–14). For example, age-related differences have been observed in the neuroscientific fields of self-awareness and self-regulation (15). It has been proposed that during normal adolescent development there is a change in the attribution of relevance to stimuli mediated by the amygdala and the amygdala input to connected functional neural networks is destabilized and re-structured to allow for an age adapted functional organization. However, in the case of adverse life events and exposure to trauma and toxic stress the same mechanisms seem to lead to dysfunction in key emotion regulation networks (16–20). Furthermore, the advances made in the scientific field of neurodevelopment and neuroplasticity have thus far not been translated into clinical applications that have yielded efficacious treatment methods for depression in adolescents and young adults.

Importantly, the division between adolescent psychiatry and psychiatry in young adulthood is arbitrary and does not match the brain maturation process (21). Successful emotion regulation and cognitive reappraisal require the coordination of multiple high-level processes (22) and structural brain development in brain regions subserving emotion regulation continues into the mid-twenties (23). The development of the prefrontal cortex is particularly protracted, with development in gray matter volume, density and thickness continuing well into the third decade of life (21, 23). In depressed adolescents the fronto-limbic brain maturation processes are delayed as compared to non-depressed individuals (24). This implies that not only adolescents, but also young adults with depression should be targeted with age-adapted treatments.

A novel neuro-scientifically based 12-week intervention, Training for Awareness Resilience and Action (TARA) was developed at University of California San Francisco and the theoretical framework that informs the intervention has been previously described (25). In summary, the program is built on and arranged within the National Institute of Health Research Domain Criteria (RDoC) framework (26) to address the major domains of function involved in adolescent depression, taking into account fundamental neurobiological and developmental aspects. The intervention is organized in a progressive manner that gives priority to the domains thought to be driving the psychopathology. First, amygdala hyperreactivity is targeted through practices promoting increased vagal afference, then training of interoceptive awareness is emphasized, followed by practices of metacognition, kindness, care and compassion for oneself and others, and finally value-based behavioral activation. The participants own understanding of the cause of their suffering is investigated and symptoms of anxiety and depression are reframed and contextualized as understandable responses to dysfunctional, stressful, or threatening environments. TARA has been shown to be feasible in both adolescent clinical and non-clinical US populations and effectively targeted depressive symptoms in a preliminary study of clinically depressed adolescents (27). Preliminary data from mixed clinical and community samples indicate that postulated brain-changes are achieved in response to treatment (28, 29). We have conducted a pilot study on Swedish medical students with non-clinical levels of stress-related symptoms to test the translated and culturally adapted TARA-manual (two TARA-groups, total *N* = 21) (unpublished data). A clinical pilot-study has also been conducted with adolescents and young adults with depression from a specialized Child and Adolescent Psychiatry unit (CAP) and a local Youth-Clinic (YC), in Northern Sweden (two TARA-groups, total *N* = 12) (unpublished data). A more definite test of the clinical effectiveness of TARA in the treatment of MDD/PDD in adolescents and young adults remains to be conducted in a two-armed randomized controlled trial, comparing TARA to standard treatment.

The increased all-cause mortality observed in adolescent MDD is mainly attributable to suicide and injuries, however, hazard ratios are also increased for a number of subsequent somatic disorders, including cardiovascular- and neurodegenerative disorders, and for death by natural causes (by a factor of two) (30, 31). Furthermore, the increased premature mortality in adult MDD is primarily attributable to general medical conditions and natural causes of death (32). Further clarity is needed with regard to the evolving field of bio-indicators in depression (33), which may inform both theory and therapy and potentially help mitigate the increased mortality. To be able to study potential bio-indicators that can elucidate the pathophysiological mechanisms involved we will therefore collect blood- and hair-samples for analysis in both study arms. Furthermore, both adolescents (34) and young adults (35) with MDD demonstrate autonomic dysregulation indicated by reduced heart rate variability (HRV) (34). Therefore, given the explicit focus on autonomic self-regulation in TARA, we will measure HRV in both study arms to test hypotheses of increased parasympathetic activity in response to TARA. Additionally, three-axial accelerometry will be collected from a randomly selected subsample to evaluate sleep and physical activity. Finally, brain Magnetic Resonance Imaging (MRI) will be collected from a randomly selected subsample, to further investigate the morphological and functional effects of TARA and standard treatment on implicated neurocircuitry.

This study will address several critical research gaps: First, we now have sufficient evidence of feasibility and preliminary efficacy after several pilot-study iterations, to progress to the next stage of the treatment development process: a randomized controlled trial. In other words, there is preliminary evidence suggesting that TARA has the potential to significantly reduce the symptom-burden in depressed adolescents and young adults, both in specialized child and adolescent psychiatry settings and primary care settings and a more definite evaluation of relative effectiveness is warranted. Second, even though it is often implied in grant proposals and research publications, as well as in the structural organization of healthcare systems, there is no such thing as a biological cut-off at the age of 18 in terms of depressive pathophysiology. Very few studies have been conducted that cover the critical age-range of 15–22, when some of the most sophisticated high-order neurodevelopment occurs (23). Third, depression trials using a more refined and diverse approach to outcome assessment that extends beyond 3-month follow-up and assesses patient-centered secondary outcomes have been warranted (36). Fourth, both published and unpublished data suggests that TARA is efficacious through its hypothesized mechanisms of action (28, 29). Mechanistic research will inform both theory and treatment development and may produce a simpler and more cost-effective approach both to treating depression and preventing relapse. Fifth, online delivered treatment is requested and preferred by many health-care users these days and online delivery is expected to increase accessibility even beyond the COVID-19 pandemic. Finally, the ISRCTN- and Clinicaltrials.gov registers (as of January 2021) record no comparable recent nor ongoing trials anywhere in the world. No current trials of depression in adolescents and young adults comprehensively speak to mechanisms of change.

The primary aim of this study is to investigate the clinical effectiveness of TARA for adolescents and young adults with MDD/PDD using a Randomized Controlled Trial (RCT)-design, with an active control based on standard treatment in specialized Child and Adolescent Psychiatry (CAP) and local Youth-Clinics (YC). Secondary aims include the investigation of dose-response relationships as well as mediating effects of emotion regulation, psychological flexibility and sleep, on treatment effect in the TARA-arm and finally, what effect the two treatment paradigms have on different bio-indicators of depression, such as heart rate variability, brain structure and function in implicated neurocircuitry, and markers of inflammation, cellular aging and stress in blood and hair. Qualitatively, we aim to explore the contextual factors perceived to drive the onset and maintenance of depression and the extent to which the different treatments are perceived to address these factors. We hypothesize that (1) TARA will result in greater reduction of self- and clinician-rated depression symptom severity at T_1_ and group-differences will be maintained or increased at T_2_ and T_3_, (2) the treatment effect of TARA will be mediated by improved emotion regulation, psychological flexibility and improved sleep, (3) reduction of depressive symptoms will correlate with improvements on bio-indicators of depression (for details please see “Outcome measures” below), and (4) the qualitative data will increase our understanding of the subjective experiences of adolescents and young adults with depression and aid further improvement and adaptation of the content and delivery of TARA and/or standard treatment.

## Materials and Methods

### Design

The study is designed as a partially nested, multi-center, parallel-group RCT with two treatment arms: (1) TARA and (2) standard treatment as recommended in national and local clinical guidelines, including multiple treatment conditions alone or in combination. Participants are randomized to either one of the two interventions, delivered *via* the local CAP and YC unit where that particular participant is seeking care or being treated. Primary outcome measure is self-reported depression symptom severity using RADS-2 total score at T_1_. The study is planned in accordance with the Standard Protocol Items: Recommendations for Interventional Trials (SPIRIT) (37) and will be analyzed and reported in accordance with the recommendations in consolidated standards of reporting trials (CONSORT) (38), with special care taken to meet extended recommendations for both pragmatic- (39) and non-pharmacological trials (40).

### Protocol Design and Conduct

We strive for full transparency throughout the study and any changes made to and/or deviations made from the protocol will be reported. Any modifications of the protocol including changes of study objectives, study design, participant population, sample size, study procedures, or significant administrative aspects will require a formal amendment to the protocol. The same applies to any changes of the protocol that may be a potential benefit, disadvantage or safety concern for the participants. Such amendments will be agreed upon by the study group, approved by the national ethical review board prior to implementation and notified to all study personnel involved.

### Center and Participant Recruitment Procedure

The study will start in the spring of 2021 and the last post-treatment measurements that will be included in the primary manuscript are expected to be collected in 2025.

Recruitment, treatment and data collection will be conducted in three to four cities in Northern Sweden, each with 1–2 centers. Several centers will be included stepwise in the RCT, until we have reached sufficient recruitment capacity. Before including a new center, we will ensure the implementation of Good Clinical Practice guidelines, the establishment of a clinical research infrastructure and the training of study-staff. A pilot trial will have been conducted at each center before center-inclusion, to show feasibility. We intend to recruit participants from the Child and adolescent psychiatry (CAP) specialized outpatient academic unit and the youth outpatient community clinic (YC) in the university city Umeå (population 89,000), CAP and YC in Skellefteå (population 36,000) and CAP in Örnsköldsvik (population 33,000). The CAP unit in Sundsvall (population 55,000) is also prepared for participation, this center will be activated if needed to maintain sufficient recruitment pace. Depending on the general recruitment rate and the feasibility of implementation of the study protocol at the different centers, one or several of them may be omitted.

Study participants will be recruited through three possible pathways: (1) by the clinical assessment team at the time of an incoming referral, (2) by clinical staff recruiting potential participants at their first clinical visit, from those wait-listed for treatment, or during ongoing standard treatment, and (3) by participants themselves responding to flyers posted in the clinics' waiting rooms or at the student health clinic at the University of Umeå. The same recruitment strategies will be used at all participating centers. Adolescents and young adults who agree to participate will be contacted over telephone for further information about the study and at that time an initial assessment for eligibility will be performed. For further eligibility assessments, please see below. For adolescents that cannot be reached over the telephone, parents or legal guardians will be contacted.

### Eligibility

Adolescents and young adults (of 15–22 years of age) that are referred to one of the specialized CAP-clinics or who are currently patients at one of the CAP or YC units and have a diagnosis of MDD or PDD according to the Diagnostic and Statistical Manual of mental disorders−4th edition (DSM-IV) or 5th edition (DSM-5) will be eligible. The clinical diagnosis for participants aged 15–17 will be validated by the Mini International Neuropsychiatric Interview for children and adolescents (41). For participants aged 18–22 the Mini International Neuropsychiatric Interview (42) will be used. Please see “Baseline measures for eligibility” for instrument details. If these measures are inconclusive the clinician rating on Children's Depression Rating Scale—Revised (CDRS-R) will be used for inclusion and a cut-off score of >40 will be used to define eligibility. For participants in CAP units that are below the age of 18 one parent/legal guardian must be available and agree to participate in parts of the sessions if the individual is randomized to the TARA-arm. Clinician interviews will also be performed to rule out exclusion criteria.

Exclusion criteria are: (1) Having one or several severe psychiatric comorbid diagnoses that may interfere with or hinder group participation, including: intellectual developmental disorder, severe autism spectrum disorder, psychotic disorder, bipolar disorder, severe anorexia nervosa, substance use disorders, severe posttraumatic stress disorder (PTSD), and severe dissociative syndromes. (2) Having one or several psychiatric symptoms or behavioral problems that may interfere with or hinder group participation, including: severe self-harming behavior, acute suicidality, a reported suicide attempt in the last 6 months or hospitalization for suicidality in the last 6 months, disabling dissociative symptoms or > 6 points as mean item score on the Adolescent Dissociative Experiencing Scale, frequent use of recreational drugs (a urine drug screen will be performed at baseline and if positive, a second test has to be negative for inclusion), reports of manic or hypomanic symptoms during the last year. (3) Having a first degree relative with bipolar disorder, since a first episode of MDD may be an incipient bipolar disorder, which is not the treatment target for TARA. (4) On-going trauma, neglect, abuse or domestic violence or destabilizing legal processes. (5) Pregnancy. (6) Non-fluency in oral and written Swedish, since the TARA groups are held in Swedish and assessment forms are in Swedish.

Other psychiatric comorbidities such as e.g., ADHD, anxiety, high functioning autism spectrum disorder and mild to moderate eating disorders are not considered exclusion criteria. Unexpected findings of biochemical anomalies at T_0_ (please see “Assessments” below) will not be considered exclusion criteria and medical conditions will be referred for appropriate treatment.

Ongoing antidepressant medication at study start will be allowed. Participants who are currently on any antidepressant medication such as Selective Serotonin or Norepinephrine Reuptake Inhibitors SSRIs/SNRIs will, if randomized to TARA, be offered a 2–4 weeks deprescription before starting TARA. If a slower tapering regimen is necessary, it will be performed alongside the initiated TARA-sessions. A senior physician will be available for consultation and for deprescription of antidepressant medication before or during TARA. Participants choosing not to discontinue medication and participants who are not successfully deprescribed will not be excluded from TARA-participation, nor from the final analysis. Medication will be included and controlled for in the sensitivity analysis.

The decision to include a participant in the study will be made by a study-clinician on the basis of the above stated criteria. In case of uncertainty the PI makes the final decision. Importantly, the enrolling clinicians will be masked to future treatment allocation, since randomization takes place at a later time, when baseline and T_0_ assessments have been completed. Having received both oral and written information about the study, participants will provide written informed consent before inclusion. For participants between 15 and 17 years of age parents/legal guardians will also provide written informed consent. Study participation is voluntary and can be canceled by the participant at any time.

### Randomization

When six eligible participants have been recruited at a study-center, they will undergo T_0_ assessments within a period of 2 weeks. Next, they will all simultaneously be randomized as a group to one of the two study arms with a 1:1 allocation ratio, by a computer-generated allocation sequence using permuted blocks and stratifying for center. The block sizes will vary randomly between two and four and the first value on the randomization list at each center will be discarded at random with 50% probability to reduce the risk of breaking the allocation concealment. Codes will be used to increase information confidentiality and participant anonymity. Treatment allocation will be performed on the basis of these codes, by a different team at the clinical research center (CRC) in Umeå. The CRC will not release the randomization results until the participants have been recruited into the trial and all baseline and T_0_ assessments have been completed. The process will thus be separate from the patient enrollment process at the clinics. Randomization will continue until *N* = 67 participants have been allocated to each treatment arm. Participants, TARA-facilitators and standard treatment personnel and clinicians delivering any co-interventions to TARA participants will not be masked to treatment allocation. However, clinical outcome-assessors and statistical analysts will be masked.

### TARA Intervention Protocol

The conceptual framework that informs the TARA intervention as well as a detailed description of the content of each session has been thoroughly elaborated (25). The treatment is framed within the Research Domain Criteria (RDoC)-matrix (26), with the addition of developmental and contextual considerations and adaptions. The TARA-intervention is organized in a systematic and progressive manner, prioritizing the major domains of function driving the psychopathology in adolescent depression.

TARA will be fully online-delivered. The intervention consists of 12 consecutive weekly online sessions, lasting 90 min each, in a group of six participants. The first session aims at creating a sense of safety by introducing the group members and facilitators; establishing clear guidelines; investigating attitudes, opinions, and previous experiences of group processes; explaining the format and introducing contemplative practices. During all sessions, participants use yoga mats for the skills-training part of the session. The two facilitators are individually signed in to the online platform. For participants that do not have access to a computer or tablet, the team will provide a tablet that is returned at the end of treatment. First, facilitators “open the circle” by ringing a bell and briefly check-in with everyone. Yoga-based movement follows, consisting of a flow of positions synchronized with the breath. Next, participants are guided through gentle breathing practices and a guided meditation practice that focuses mainly on interoceptive and sensory awareness that ranges from 1 min in the first session to 15–20 min duration toward the end of the 12-week intervention period. After a short break there is a brief psychoeducational presentation followed by group exercises and discussions. Then there is time for feedback and questions regarding the home practice from the previous week and the participants are walked through the practices for the coming week. Finally, the session ends by a “closing of the circle,” when the participants gather their attention and have the opportunity to express any reflections or thoughts regarding the session. If a school holiday occurs during the 12-weeks, treatment can be paused for up to 4 weeks. In such cases the treatment period will be extended, so that a total of 12 sessions are still given.

The 12 sessions are divided into four modules, each with specific pathophysiological and neurophysiological targets. In module one: *Calming Down and Creating a Sense of Safety* (sessions one to three), the target is to reduce the amygdala hyperreactivity to emotional stimuli and to achieve this the participants learn breathing practices and yoga-based movement. In module two: *Attending to and Caring about Our Inner Experience* (sessions four to six), the target is to shift neural activity away from the brain's default network by engaging the salience network, including the anterior insular cortex. This is done by the practice of noticing negative self-referential processing and then to intentionally shift to present moment sensory awareness. To achieve this, interoceptive attention and emotion labeling is also practiced. In Module three: *Recognizing, Regulating*, and *Communicating Emotions* (sessions 7–9), the target is to use the skillset acquired from previous modules to regulate emotions and reduce interpersonal stress during social interactions. Participants practice recognizing emotional triggers in oneself and others, empathetic listening, effective communication, and compassionate responses to oneself and others. In module four: *Core Values, Goal Setting*, and *Committed Action* (sessions 10–12), the target is to increase behavioral activation guided by intrinsic reward. This module is partly inspired by techniques in acceptance and commitment therapy (43) and here participants train to recognize and address their own experiential avoidance in order to identify and then be guided by their core values when they take action in their lives. The goal is also to be able to apply cognitive control over emotional experiences by using the previously acquired skillset while engaging in their own social networks and community. Symptoms of depression and anxiety are contextualized and addressed from a systemic perspective.

The development of TARA was inspired by the structure and content of mindfulness-based approaches, TARA is however fundamentally different in several ways. First, manipulation of the breath and synchronized slow movements are used to improve emotional self-regulatory skills, rather than primarily focusing on acceptance of emotional experience through metacognition. Second, there is a focus on “real world” relevance for the adolescents and young adults, and transgenerational dialog and inquiry is emphasized. Third, full transparency is aimed for, by giving the explicit rationale for each practice, often with a scientific background explained in the psychoeducational presentations. Fourth, interaction and relational practices are emphasized, as well as practices of validating and skillfully expressing emotional experiences. Fifth, value-based committed action is an extended goal of the curriculum, not only equanimity and personal well-being. Finally, time is spent contextualizing depressive symptoms and investigating potentially negative impacts that the cultures and systems we live in may have on personal health, according to Bronfenbrenner's socioecological theory (44).

Home practice of TARA skills is encouraged and audio tracks with instructions for breathing and movement, as well as short, guided, meditations are provided to all participants. Short video recorded summaries of the session content presented by the principal investigator will also be provided to support the skills uptake.

A short meeting is offered to all participants/parents/legal guardians before starting the program to resolve potential technical issues related to the online platform. For TARA-group participants in the CAP-clinics, that are below 18 years of age, parents/legal guardians will be offered a short session in conjunction with the initial TARA group session, and again after session six and 11, to be able to better support the participants between the sessions. For those participants, one parent/legal guardian or other important adult is also invited and expected to participate in the first skills-training part of each session. If necessary, participants may also be offered one individual preparatory session before session one, as well as one or two booster sessions with one of the facilitators throughout the program. A concluding gathering will be offered to all participants for further instructions regarding follow-up.

Participants who do not show up for sessions will be contacted over the phone the following day to discuss the reasons for not participating, to assess their status and safety, and to support their home practice. If they cannot be reached themselves, parents or legal guardians will be contacted in the case that the participant is under 18 years old. Extra individual meetings with one of the facilitators may be offered online between the TARA sessions, for example if a session is missed and clarification or extra support is needed. The number of such sessions will be reported with descriptive statistics. If any new prescription of psychotropic medication or additional treatment such as hospitalization is required, the participant will still be offered continued TARA group participation if possible. This is not a part of the TARA intervention protocol and will therefore be reported as protocol non-adherence according to the description under “Adherence to protocol” below.

Each session for a given group is facilitated by the same two TARA-trained facilitators, each with experience from clinical child-and adolescent psychiatry or psychiatry and contemplative practice. Their professions will include psychiatrists, resident physicians, clinical psychologists, social workers and physiotherapists. Facilitators will be allocated to each trial group by the principal investigator and the directors of the different centers based on local availability. Fidelity to the TARA manual, both in terms of adherence to content and the process of delivery, is assessed formally in four random sessions out of 12 by the use of a specific TARA-fidelity scale. This is done either by an observer who participates online during the sessions or by the assessment of audio recordings of the sessions. Non-fidelity to the manual is managed by feedback to the facilitators and adjusted in the following sessions. Ongoing supervision and feedback will be provided. The facilitators' task during the sessions is not only to teach specific content, but also to model a collaborative, inclusive, non-judgmental, and supportive attitude.

### Standard Treatment Protocol

Participants allocated to the control condition receive standard treatment based on national and local guidelines, availability and the clinician's and patient's preferences. For outpatient treatment of MDD and PDD in adolescents the Swedish National Board of Health and Welfare guidelines recommends psychoeducation as first line treatment, and for non-responders', in mild to moderate cases, additional cognitive behavioral therapy and in moderate to severe cases the SSRI Fluoxetine is recommended. All these modalities are given equally high priority. Adult guidelines for outpatient treatment include psychotherapy, physical activity or antidepressant medication alone or in combination, given different priority according to symptom severity (45, 46). In our pragmatic study we will not control which specific treatments are given in the standard treatment arm, nor the combination, frequency or timing of them. Instead, we aim to compare TARA delivered as described in this protocol to the standard treatment as it is actually delivered today. All physician-prescribed treatment and concomitant care interventions will be allowed in this study arm. Standard treatment will be delivered online or in person/groups according to local routines. To describe the treatment content in the standard treatment arm, medical records will be assessed retroactively.

### Adherence to Study Protocol

We will retroactively collect data from the medical records of all participants at T_2_, including information on psychiatric diagnoses, time to treatment initiation, treatments offered (psychological, pharmacological, TARA, inpatient care, and other), number of clinical visits/sessions offered and attended, as well as any change of and termination of treatment. A descriptive summary of this data will be reported categorized into T_0_-T_1_ and T_1_-T_2_ time periods.

This way, full details of any additional (non-randomized) treatment will be provided. The information will also allow for the values of any outcome data collected after treatment withdrawal to be put into context and will enable a more detailed description of the participants lost to follow-up.

Protocol non-adherence in the TARA-arm is defined as (1) Facilitators repeatedly not adhering to TARA-manual and not promptly correcting their fidelity to the manual upon receiving feedback, (2) Introduction of concomitant psychotropic medication and/or psychotherapy that is not included in the TARA intervention protocol, (3) Participant missing more than 50% of TARA-sessions, and (4) The monitoring performed by the CRC, for details please see “Data and safety monitoring” below, reveals any major protocol violation, which in such cases will be elaborated in detail.

Protocol non-adherence in the standard treatment-arm is defined as (1) Participant non-adherence to medication stated in the participant's medical records, (2) participant missing more than 50% of offered clinical appointments. In case no treatment is offered at all, that will not be considered a case of protocol non-adherence, and (3) The monitoring performed by the CRC, for details please see “Data and safety monitoring” below, or the medical record analysis at the end of the study reveals any major protocol violation, which in such cases will be elaborated in detail.

All outcome measures, please see “Assessments” below, will be collected from participants also in cases of protocol non-adherence. All data will be included in the main intention to treat analysis and cases of protocol non-adherence will only be excluded from per-protocol analysis, for details please see “Statistical methods” below.

### Adverse Events and Security Plan

Adverse events (AEs) are defined as any undesirable experience requiring healthcare occurring to a participant during the study, whether or not considered related to treatment. All AEs reported spontaneously by the participant or observed by the investigators, standard treatment physicians/therapists and TARA facilitators as well as those encountered at medical record analysis will be recorded in the electronical case report form. All AEs and serious adverse events (SAEs, resulting in death, life threatening, requiring hospitalization or other important medical events) will be communicated to the principal investigator. SAEs that result in death or are life threatening and are potentially causally related to the study will be reported to the accredited ethics committee that approved the protocol within 7 days of the responsible researcher first learning of the SAE. Participants who experience AEs/SAEs and as a result of that decide to discontinue their participation in the study will still be included in the main intention to treat analysis.

Based on previous studies of TARA (27–29) and unpublished preliminary data, deterioration of depressive symptoms during TARA is not expected. However, for safety reasons session-by-session assessments will be performed, using Outcome Rating Scale and Session Rating Scale, for details please see “Session by session assessments” below. Before each session the participants will also complete a brief report of any AEs during the last week that has required seeking healthcare. Facilitators will also be observing any adverse reactions in the TARA-groups and deterioration of symptoms will be discussed with the participant and their parents or legal guardians if adequate.

In the standard treatment arm any deterioration of symptoms during the treatment will be handled by the clinic through their standard procedures. Since treatment in both study arms is given as a part of the regular health care system, all participants are covered by the patient injury insurance.

### Assessments

Pre- and post-treatment self-report scales will be administered using an online platform to which only the research group has access and coded data will be stored on a secure server. All data from other assessments will be entered and stored on the same secure platform. Biological specimens obtained will be stored coded in a certified biobank for batch-wise analysis once the dataset is complete. Additionally, at the T_0_ blood-draw we will follow a clinical standard protocol for routine depression-diagnostics and analyze depression related biochemical parameters that can be indicative of underlying somatic disease. The outcome of these analyses will not be reported unless frequent or otherwise significant deviations appear, rendering their reporting necessary for the final result-interpretations.

Participants will receive reimbursement equaling approximately five USD each time they complete assessments and 10 USD for each blood-draw at T_0_ and follow-up. Participants who do not show up for appointments or complete assessments will be reminded by email or telephone. A schedule of the enrolment, interventions and assessments is provided in Table 1.

**Table 1:** Schedule of enrolment, interventions, and assessments.

	**Study period**						
	**Enrolment**		**Allocation**	**Post allocation**			**Close-out**
**Timepoint**	**T_**−1**_**	**T_**0**_**		**T_**0.5**_ (1.5 m)**	**T_**1**_ (3 m)**	**T_**2**_ (6 m)**	**T_**3**_ (24 m)**
**Enrolment**							
Eligibility screening	X						
Informed consent	X						
Clinician assessment	X						
Allocation			X				
**Interventions**							
TARA			I 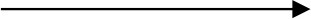 X				
Standard Treatment			I 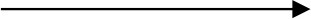 X				
**Assessments**							
Self-report	X	X			X	X	X
Clinician rating	X				X		
Blood sampling		X			X		
Hair sampling		X			X		
Heart rate variability		X			X		
Accelerometry (not all)		X		X	X		
Magnetic Resonance Imaging (not all)		X			X		
Qualitative measures (not all)		X			X	X	
Medical record analysis						X	
**ASSESSMENTS (TARA-arm only)**							
Self-report				X			

#### Baseline Measures for Eligibility

*Mini International Neuropsychiatric Interview* is a short structured diagnostic interview for DSM-IV and International Classification of Diseases 10th revision (ICD-10) psychiatric disorders that has now been updated for DSM 5. With an administration time of ~15 min, it was designed to meet the need for a short but accurate structured psychiatric interview for multicenter clinical trials and epidemiology studies and to be used as a first step in outcome tracking in non-research clinical settings (42).

*Mini International Neuropsychiatric Interview for children and adolescents* is a short structured diagnostic interview for DSM-IV and ICD-10 psychiatric disorders in children and adolescents (41). Mini International Neuropsychiatric Interview for children and adolescents disorder classifications have shown test-retest reliability and validity comparable to other standardized diagnostic interviews and is claimed to be a useful tool for diagnostic screening in Child and Adolescent Psychiatric care (47).

*Children's Depression Rating Scale—Revised* is a standardized rating scale based on a semi-structured interview and provides an efficient way to diagnose childhood depression and to monitor treatment response (48). A cut off score of >40 will be used for eligibility in case other assessments are inconclusive. In cases when the trained assessor is not a specialist in child- and adolescent psychiatry the assessments will be recorded for quality control.

The *Adolescent Dissociative Experiences Scale* measures dissociation and is validated in Swedish adolescents. The 30 items cover dissociative amnesia, absorption and imaginative involvement (including confusion between reality and fantasy), depersonalization, derealization, passive influence/interference experiences, and identity alteration (49). Total score range is mand mean item score is 0–10, higher scores indicate more dissociation.

Urine samples will also be collected and analyzed for drug-metabolites, if positive a second test has to be negative for inclusion.

#### Baseline Measures for Description of Sample

The *Social Support Questionnaire* is a 13-item form with questions targeting social support, the lack of which is a proximal risk factor for depression (50).

The participants *sociodemographic background* will be assessed using a brief self-made questionnaire.

*Childhood Trauma Questionnaire* will be used to screen for adverse childhood events. This 28 item self-report measure assesses history of emotional abuse and neglect, physical abuse and neglect and sexual abuse. Reliability and validity in seven studies with clinical and non-clinical samples with over 2,000 respondents have demonstrated satisfactory to high internal consistency (Cronbach's alpha of 0.95) and robust construct validity, with psychiatrically referred groups reporting higher levels of abuse and neglect than non-clinical samples (51, 52).

The *Adverse childhood experiences* is an internationally well-used and validated screening interview covering physical, sexual and emotional abuse, physical and emotional neglect, exposure to domestic violence, household substance abuse, and mental illness, parental separation/divorce or incarceration of household member. Each item is assessed as zero or one, with a total score ranging from 0 to 10 (53).

#### Outcome Measures

Outcome measures are collected at T_0_, T_1_, T_2_, and T_3_. Data collection for some of the outcome measures is only performed at some of the timepoints. Please see each respective subheading and Table 1 for details. For participants that miss a particular data collection timepoint we will apply the following measures sequentially: (1) automatic reminder(s) sent by email, (2) SMS reminder, and (3) telephone contact with the participant/parent/legal guardian.

##### Primary Outcome Measure

As primary outcome measure, we will use self-reported depression symptom severity with *Reynolds Adolescent Depression Scale 2nd edition* (RADS-2) (54) total-score at T_1_. The RADS-2 has excellent psychometric properties and is validated in adolescents with depression (54). It contains 30 items, providing four subscales measuring different dimensions of depression: Dysphoric Mood, Anhedonia/Negative Affect, Negative Self- Evaluation, and Somatic Complaints. Raw scores range from 30 to 120 and higher scores indicate more severe depression symptoms. The scale has been translated to Swedish and validated in a Swedish adolescent general population (55). Validation in a Swedish clinical population is currently being undertaken by our group (unpublished data).

##### Secondary Outcome Measures

*Self-Report:* As a secondary outcome measure, we will use RADS-2 total-score at T_2_.

Additional self-report will be performed at T_0_, T_1_ and T_2_ using *Multidimensional Anxiety Scale for Children*. Anxiety disorders are highly comorbid with MDD in adolescents and the Multidimensional Anxiety Scale for Children is considered the best normed and psychometrically strong self-report anxiety scale to use in adolescents (56, 57). Total raw score range is 0–117 and higher scores indicate more severe anxiety.

*Clinician Rating:* We will perform clinician rating of depression symptom severity by a trained clinician masked to treatment allocation using Children's Depression Rating Scale - Revised (CDRS-R) at T_0_ and T_1_. CDRS-R is a standardized rating scale based on a semi-structured interview and provides an efficient way to diagnose childhood depression and to monitor treatment response (48). Raw scores range from 17 to 113 and higher scores indicate more severe depression. In cases when the trained assessor is not an experienced CAP-physician the assessments will be recorded for quality control.

##### Other Outcome Measures

*Self-Report:* The self-report scales RADS-2 and *Multidimensional Anxiety Scale for Children* described above will also be collected at T_3_. All self-report scales below will be collected at T_0_, T_1_, T_2_, and T_3_.

The *Montgomery–Åsberg Depression Rating Scale—Self Assessment* (MADRS-S) (58) is a nine-item self-report depression scale and is adapted from the clinician rating scale MADRS (59). MADRS-S includes questions on the following symptoms (1) Mood, (2) Inner tension, (3) Sleep, (4) Appetite, (5) Concentration difficulties, (6) Lassitude, (7) Inability to feel, (8) Pessimistic thoughts, and (9) Suicidal thoughts. Items are rated to capture the participant's depressive state over the past 3 days. In the clinician rating scale, both reported and apparent mood are rated, apart from that the two scales are identical. On the self-report version each item yields a score of 0–6 and total score range is zero to 54. Higher scores indicate more severe depression.

The *Multiscale Dissociation Inventory* is a 30-item self-report measure of dissociative symptomatology. It is standardized and normed in a U.S. population (60), and measures six different types of dissociative response. We have translated the scale and are currently conducting validation studies in Sweden. Total score range is 30–150 and higher scores indicate more severe dissociation.

The *Negative Effects Questionnaire* is a 32-item scale validated in Swedish (61). It measures negative effects of psychotherapy and will be collected from TARA-participants and all standard care participants receiving some kind of psychological treatment. First, respondents endorse specific items in case they have occurred or not during treatment, with a yes or no. Second, the respondents rate how negatively the negative effect was on four-point Likert-scale, ranging from “Not at all” to “Extremely.” Third, the respondents attribute the negative effect to “The treatment I received” or “Other circumstances.” There is currently no consensus on how to best interpret scores from this measure. However, summing up the frequencies and providing information on means and standard deviations can help with comparisons between the samples.

The *Pediatric Side Effects Checklist* is a comprehensive checklist identifying many different potential side-effects of medication. It will be collected from all participants who are taking psychotropic medications. The checklist was developed by Pavuluri et al. (62) and has since been translated to Swedish by Bengtsson Macri.

The *Insomnia Severity Index* is a seven-item scale that is considered a reliable and valid instrument to detect cases of insomnia in this population and is sensitive to treatment response in clinical patients (63). Total score range is 0–28 and higher scores indicate more severe insomnia.

The *Perceived Stress Scale:* Perceived stress will be assessed by the well-validated ten-item version of this scale (64). Total score range is zero to 40 and higher scores indicate more severe stress.

*Avoidance and Fusion Questionnaire for Youth* is an eight-item measure of psychological flexibility rating: (a) Cognitive fusion; (b) Experiential avoidance; (c) Inaction or behavioral ineffectiveness in the presence of unwanted internal experiences (65). The scale has been validated in Swedish adolescents (66, 67). Total score range is 0–32 and higher scores indicate less psychological flexibility.

The short version of *Difficulties in Emotion Regulation Skills* (68) will be used to measure emotion regulation skills. Total score range is 16–80 and higher scores indicate less emotion regulation skills.

The *Compassionate Engagement and Action Scales—youth* measures different aspects of compassion which have been shown to be related to depressive symptoms. The three subscales measure compassion to self, compassion to others, and experiencing/being aware of the compassion from others. Each scale can be analyzed in terms of the engagement and action aspects separately or as a single factor (69). A youth-version of the scale has been validated in Swedish adolescents (70). Total score range is zero to 270 and higher scores indicate more compassionate engagement and action.

*Strength and Difficulties Questionnaire* is a 25-item questionnaire well-validated in Swedish that assesses the psychological adjustment of children and youths. The items are divided between five scales consisting of five items each; emotional symptoms, conduct problems, hyperactivity-inattention, peer problems, and prosocial behavior (71–73). Total score is calculated by summing up the scores of the first four subscales, the total range is 0–40 and higher scores indicate more difficulties. The fifth subscale prosocial behavior has a range from 0 to 10 and lower scores are interpreted as indicative of a problem.

*Euro Quality Of Life*−*5 Dimensions—Youth* is a general quality-of-life instrument designed to measure health-related quality of life in children and adolescents. It consists of five items and a visual analog scale and has been validated in Swedish clinical adolescent populations (74, 75). Total score range on the five items is five to 15 and higher scores indicate lower quality of life.

*Deliberate Self Harm Inventory* (DSHI-9r) adapted to adolescents (76, 77) is an instrument measuring deliberate engagement in any of nine different forms of self-harm during the past 6 months, with responses ranging from “never” to “more than five times.” The DSHI-9r shows good test–retest reliability (77). Total score range is zero to 54 and higher scores indicate more self-harm.

The *Suicidal Ideation Questionnaire—Junior* is a 15-item scale designed to assess the severity and frequency of suicidal ideation among adolescents (78). Validation in the intended population is currently being undertaken by our group. Total score range is zero to 90 and higher scores indicate more suicidal ideation.

The *Emotional Breakthrough Inventory* is a six-item scale that assesses the presence and resolution of emotionally challenging/distressing experiences that can occur during therapeutic depression treatment. The scale utilizes visual analog responses captured on a line from not at all to very much so. The rated experiences include (1) facing emotionally difficult feelings that are usually pushed aside; (2) experiencing a resolution of a personal conflict/trauma; (3) being able to explore challenging emotions and memories; (4) having an emotional breakthrough; (5) getting a sense of closure on an emotional problem, and (6) achieving an emotional release followed by a sense of relief. It is validated in adults with depression (79) and the questions seem particularly relevant given the therapeutic approach in TARA. Validation in the intended population is currently being undertaken by our group. Total score range is zero to 100 and higher scores indicate a higher degree of emotional breakthrough.

The *Patient-Reported Outcomes Measurement System* is a set of person-centered measures developed using item response theory that evaluates and monitors physical, mental and social health in adults and children (80). We will use the anger and physical activity item banks. Higher scores are equivalent to more of the construct being measured, for the anger item bank a higher score indicates more anger and for the physical activity item bank a higher score indicates more physical activity. PROMIS item banks are developed to be used transformed to *T*-scores with a mean of 50 (SD 10) in US population samples. Validation in a Swedish sample is currently being undertaken by our group.

*Clinician Rating:* Children's Global Assessment Scale will be recorded by the trained clinician performing clinician-rating at T_0_, and T_1_. The scale is well-established and widely used to measure functional capacity in everyday life in children and adolescents (81). The Global Assessment of Functioning is the equivalent scale used for adults. We will collect clinician rating on these two scales for descriptive purposes, they will however not be analyzed in formal hypothesis-testing given their questionable reliability (81). Total score range is zero to 100 and higher scores indicate a higher global functioning.

*Physiological Measures:* To measure heart rate variability (HRV) at T_0_ and T_1_ we will use medical CE-certified and FDA-registered NeXus10 MKII^1^ with bipolar ECG channels and digital sample rates up to 8,192 Hz in 24-bit resolution, using active shielding for minimal noise and movement artifacts. A 10-min registration in supine position, after 15 min of rest with no intervention will be done. No caffeine or other stimulants will be allowed for 4 h prior to registration. Processing of raw data will be done with Kubios software^2^. Meta-analysis has shown lower resting state HRV among clinically depressed children and adolescents compared to healthy controls, a finding that is consistent with findings among adults (82). Given that TARA is designed to increase vagal afference and promoting autonomic regulation an increase in HRV is considered an important secondary outcome.

Systolic and diastolic blood-pressure will be measured before the HRV-registration. At the same visit we will also measure height, weight, and waist-and hip-circumference to use this information in the metabolomic analyses (please see below).

The Motionwatch8^3^ will be used to measure triaxial accelerometry for physical activity analysis. Company-provided software include advanced tools for determining sleep efficiency, sleep fragmentation, sleep latency and many more actigraphy derived sleep parameters. The devise also has an in-built light sensor to monitor light exposure and allow for circadian rhythm analysis. Randomly selected participants from both trial arms in the Umeå YC/CAP-populations will wear the device for 2–3 consecutive weeks at T_0_, for 2–3 consecutive weeks at T_0.5_, as well as for 2–3 consecutive weeks at T_1_.

*Blood and Hair Samples:* Blood will be drawn at T_0_ and T_1_ and stored in a biobank for batchwise analyses after the completion of data collection. We will investigate bio-indicators of MDD/PDD, including but not limited to: neurotropic factors (Brain-Derived Neurotrophic Factor, Epidermal Growth Factor, Vascular Endothelial Growth Factor, Nerve Growth Factor, Glial cell-line Derived Neurotrophic Factor, Insulin-like Growth Factor-1), cytokines (interleukins IL1-a, IL1-b, IL-2, IL-4, IL-6, IL-8, IL1-0, Tumor Necrosis Factor-alpha, Interferon-gamma, and Monocyte Chemoattractant protein 1), metabolomics with a focus on circulating lipid metabolites associated with adult depression (83), and telomere length in peripheral blood mononuclear cells.

In hair cortisol levels at T_0_ and T_1_ will be analyzed.

*Magnetic Resonance Imaging:* MRI will be performed at T_0_ and T_1_ in approximately *N* = 22 randomly selected participants from each study arm in the Umeå YC/CAP-populations in the second half of the study for logistical reasons. Functional connectivity at resting state as well as task-based MRI will be performed to investigate the effects that the different treatments have on depression related areas of the brain. Morphology and structural connectivity will also be assessed. For the subsample of participants that will do MRI *additional exclusion criteria* will be applied, including having metallic implants, color blindness, left-handedness, claustrophobia, intake of any potentially mind-altering substance within 24 h prior to scanning (including any psychotropic medication and recreational drugs), and obesity if a hindrance for entering the scanner.

*Measures for Mediation Analysis:* To investigate mediating effects on treatment response we will administer the self-report scales *Difficulties in Emotion Regulation Skills, Avoidance, and Fusion Questionnaire for Youth* and *Insomnia severity index* at T_0.5_. The accelerometry and sleep parameters that will be registered in randomly selected participants at T_0.5_ in both study-arms will also be used for mediation-analysis in the TARA-arm.

*Session by Session Assessments in the TARA-Arm:* Additionally, before and after each TARA-session the Outcome Rating Scale and Session Rating Scale will be used (84). These are self-assessments using four items on a ten-centimeter visual analog scale (84), with higher scores indicating better functioning/experience. Participants will complete the Outcome Rating Scale before each session, rating how they have been doing individually, in the family, socially, and overall, during the previous week. The Session Rating Scale, a measure of working alliance, will be completed after each session, where they rate how much they felt listened to, how important the content and activities were to them, how much they liked the session, and their overall experience. A brief self-made self-report form in the TARA-arm will also be completed, in which participants report the amount of home-practice they have done each day during the last week, to enable dose-response analyses. At each session a brief checklist for any adverse events occurring in the previous week will also be administered. All these forms will be completed online.

*Qualitative Measures:* Qualitative data will be gathered in personal semi-structured interviews with ~20 purposively sampled participants. Interviews will be conducted before and/or after the completion of treatment (after 3 months), with participants from both study arms. The aim is to reach a more comprehensive understanding of the subjective experience in adolescents and young adults of the mechanisms and processes involved in the onset and maintenance of depression. We will also explore their experiences of the treatment they have received and the extent to which it addressed those factors in a relevant and meaningful way. Interviews will be recorded, transcribed and analyzed using appropriate qualitative methods. Integration with the quantitative data will enhance understanding of change mechanisms that can improve TARA's potential efficacy. An additional signed consent will be obtained from every participant recruited for this ancillary study.

### Data and Safety Monitoring

A data protection officer at the CRC independent from the investigators and from the funders will be assigned to centrally monitor: (1) the protection of the rights and well-being of the participants; (2) whether the reported research data is accurate and verifiable in source documents; and (3) accuracy and completeness of data. Monitoring will be performed according to a separate monitoring plan. The results of this monitoring will be summarized and reported to research personnel.

### Statistical Methods

#### Analysis Plan

The analyses will be performed after data collection is complete. No interim analyses will be conducted. First, data quality and completeness will be assessed and if available, missing values in the dataset will be entered from the individual's file in the original data source. We will check the dataset for outliers and illogical values. For categorical variables, responses outside the possible response categories will be coded as missing. For continuous variables, values outside the possible ranges will also be coded as missing.

Analyses will be performed by the investigators and a biostatistician using the latest version of SPSS statistics (IBM Corp., Armonk, NY, USA) or equivalent statistical software. All data analysts will be masked to treatment allocation. All significance testing will be two-tailed (with the potential exception of non-inferiority analysis, please see below) and statistical uncertainties will be expressed in 95% confidence intervals. All tests will be performed using a significance level of 0.05.

##### Descriptive Statistics

Baseline data and descriptive statistics will be reported using standard measures in accordance with CONSORT guidelines and the baseline characteristics of the included participants will be reported per randomization group in a baseline table.

The following baseline characteristics will be reported: age (years), sex (% F, % M), diagnosis (% MDD, % PDD, % recruited on CDRS-cut-off only), psychiatric comorbidity (% yes, by diagnostic categories), Adolescent Dissociative Experiencing Scale score, Childhood trauma questionnaire score, Social Support Questionnaire score, RADS-2 score at T_0_, use of antidepressant medication at T_0_ (% yes, by medication categories), use of other psychoactive medication at T_0_ (% yes, by medication categories), smoking at T_0_ (% yes, frequency), country of birth (% Sweden, % first generation immigrant and % second generation immigrant—with at least one parent born outside of Sweden), recruitment method and study center.

As significance-testing for differences in baseline characteristics between study arms is not recommended given that any differences are necessarily the result of chance rather than bias (38), it will not be performed unless specifically required by the intended journal.

The following follow-up characteristics will be reported: primary and secondary outcome measures, use of antidepressant medication at T_1_ and T_2_ (% yes, by medication categories), use of other psychoactive medication (% yes, by medication-categories).

Protocol deviations that will be summarized include those that are specified in “Adherence to study protocol” above (%, type and elaboration if necessary). Attendance will be presented as the ratio of sessions attended/sessions offered in the TARA arm and as the ratio of appointments attended/appointments offered in standard treatment. The content of standard treatment as well as any additional concomitant treatment in the TARA arm will be reported as described in “Adherence to study protocol” above. Compliance to prescribed pharmacological treatment will be assumed if not otherwise specified in the medical records.

Participants who do not show up for baseline and T_0_ assessments and participants who complete baseline and T_0_ assessment but drop out before randomization will be considered external dropouts and will not be included in the analysis. Participants who are randomized, but do not show up for treatment or show up but do not complete the allocated treatment, will be considered internal dropouts and will be included in the intention to treat analysis. All participants missing at follow-up will be contacted over the phone to clarify the causes of dropping out. We aim to enable a comparison of the reasons given for drop out between the two study arms and examine potential sources of bias in attrition. Participants who have dropped out will be asked to still contribute with data on the primary outcome. Participants will not be excluded from further follow-up and analysis in the case of protocol violation in eligibility. This means that randomized participants who are discovered to no longer qualify as eligible will still be followed-up and analyzed according to the principle of intention to treat.

##### Patient Flow Diagram

**Figure 1:**
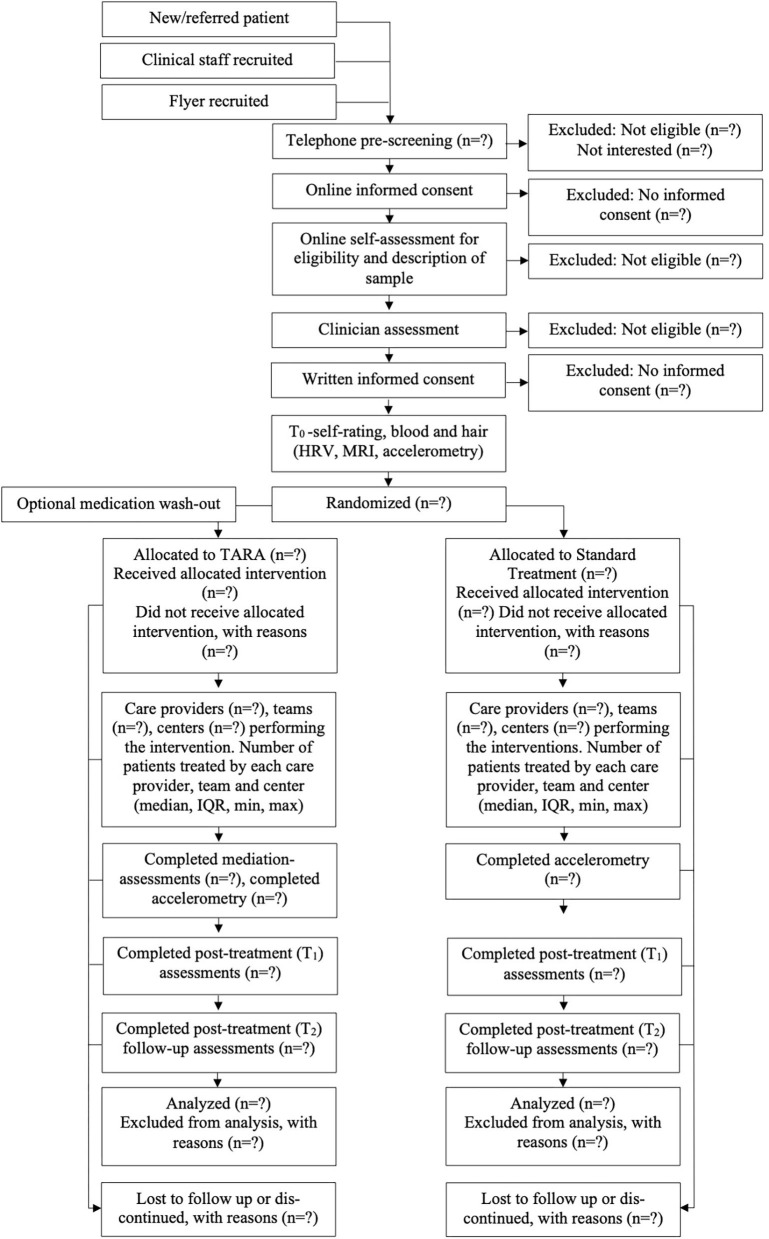
Flow of participants.

The flow of participants will be illustrated in a flow diagram according to CONSORT recommendations (Figure 1).

##### Missing Values

We will not impute missing data on the primary outcome. A simulation study has found that a mixed-effects model in repeated-measures analysis is robust in maintaining the statistical properties of a test when compared to the multiple imputation method for handling missing values (85), assuming that participant drop-out is unrelated to outcome conditioned on the study covariates (missing at random assumption). Since this is assumed by most of the currently valid techniques used for handling missing data (86, 87) and we collect measures to help increase the plausibility of making that assumption a realistic one, we will use this approach in our analysis. Frequency and pattern of missing data will be reported.

##### Analysis of Primary Outcome

All randomized participants will be included in the main analysis that will be performed according to intention to treat. The primary outcome RADS-2 total raw-score at T_1_ aims to investigate differences between the two treatment arms in reduction of depressive symptoms and will be analyzed using hierarchical mixed effects modeling to account for the clustering caused by partial nesting. The main mixed effects model will include fixed effects for treatment allocation, RADS-2-score at T_0_, age-group (dichotomous, 15–17 and 18–22 years old), study center and sex. The clustering effect will be modeled using a random intercept for each intervention group. We expect that compound symmetry will be the most appropriate choice of correlation structure for this model.

Superiority will be concluded if 95% CI:s are not overlapping between the two study-arms. Additionally, a non-inferiority margin is defined as a RADS-2 total raw-score difference of eight points. This we consider to be the maximal clinically acceptable difference in light of the other potential benefits of TARA. If superiority is not demonstrated, that is not a reliable indicator of equivalence (88, 89) and additional specific equivalence- and non-inferiority analyses will be performed (90). Switching from superiority to non-inferiority does not appear to create statistical multiplicity and therefore no multiple-comparison-correction will be made in case we perform a non-inferiority analysis (91). Since participants who withdraw or drop out may tend to have a lack of response, using the full analysis set is likely to be biased toward demonstrating non-inferiority. Therefore, a per-protocol population (defined using the definitions of non-adherence stated in “Adherence to protocol” above) will be used in this analysis. Additionally, to avoid making biased conclusions based on the per-protocol population we will also perform the analysis on the intention to treat population and conclude non-inferiority if the analyses reach similar results.

##### Analysis of Secondary Outcomes

Secondary outcome variables will be analyzed using similar mixed effects models as used for the primary outcome to accommodate for clustering effects. Time (T_0_/T_1_/T_2_) and the allocation by time interaction will be included as fixed effects in the models when analyzing variables measured at more than two timepoints. We expect that an autoregressive correlation structure will be most appropriate in these cases.

Secondary outcomes will be reported as point estimates with 95% confidence-intervals. Since statistical multiplicity does not arise when there is no opportunity to choose the most favorable outcome analyzed (92), pre-specified secondary outcome-estimates will not be corrected for multiple comparisons in the primary manuscript unless specifically required by the intended journal. There are no pre-planned subgroup analyses.

##### Analysis of Safety Outcomes

Safety outcomes are all AEs/SAEs due to complications of depression or treatment and non-trial-related AEs/SAEs, between inclusion of the patient in the trial and T_2_. The number of AEs/SAEs will be reported by their relationship as “definitely,” “probably,” “possibly,” and “unlikely” related to treatment. The number (and percentage) of participants with each AEs/SAEs will be presented for each treatment arm categorized by (1) preferred term (2), body system, (3) severity. The number (and percentage) of occurrences of each AE/SAE will also be presented for each treatment arm. No formal statistical testing will be undertaken as this would be inappropriate due to lack of power (93).

##### Analysis of Dose-Response

Dose response relationships within the TARA-arm will be analyzed using similar mixed effects models as used for the primary outcome to accommodate for clustering effects. Session attendance as well as self-reported data on the amount of each home-practice they have done for each day during the last week will be included as fixed effects. We expect that compound symmetry will be the most appropriate choice of correlation structure for these models.

##### Sensitivity Analyses

The following individual sensitivity analyses will be performed on the primary outcome to test the robustness of findings.

Missing data on repeated measurements is robustly handled by linear mixed modeling (94). To evaluate how sensitive the primary outcome result was to missing data we will use imputed values. Single value imputation-methods are generally considered naive, can introduce bias and artificially increase precision (86). Since multiple imputation provides more valid results (87) it will be used to impute missing values that will then be tested using the same model as in the primary analysis. All predictors in the main analysis and additional background covariates will be used in the imputation model. Fifty iterations will be used in Multiple imputation by chained equations and as seed we will use 49,225 which we generated as a random number between one and 100,000.

The impact of residual baseline imbalances will be investigated by residual analysis and subsequently removing sex and age-group at T_0_ from the model. The impact of clustering effects will be investigated by examining the random effects for group within center in the model. The impact of number of days with antidepressant medication will be analyzed by entering that variable as a factor in the model. The impact of protocol non-adherence will be tested by performing the analysis separately on the per-protocol population defined using criteria specified in “Adherence to protocol” above.

To examine potential sources of and impact of bias in attrition, differences in characteristics between participants lost to follow-up and participants retained in the study will be assessed.

The same sensitivity analyses will be performed in case a non-inferiority analysis is used for the primary outcome.

##### Mediation Analysis

For mediation analysis the effect of TARA on RADS-2 score will be partitioned into indirect and direct effects. Indirect effects of the variables measured halfway through TARA, for details please see “Measures for mediation-analysis” above, will be modeled using the appropriate statistical methods in causal mediation analysis since it is more rigorous than statistical mediation analysis and allows for assessment of nonlinear relationships and interactions (95). Known and available confounders will be adjusted for and additional sensitivity analyses will be performed to assess the potential bias caused by unmeasured confounders.

#### Sample Size Calculation

Sample size was calculated using R version 3.6.1 (2020, R-core team, Vienna, Austria) for achieving a power of 0.80 at a significance level of 0.05 (two-tailed) for the primary outcome, also considering specific recommendations for clustered designs (96). We assumed a partially nested cluster design with a cluster-size of six subjects with an intra-cluster correlation coefficient of 0.3 and a correlation between T_0_ and follow-up score of 0.7, based on pilot study data. In order to detect the minimal treatment effect that we consider clinically relevant, of eight raw-score points on RADS-2 (based on the disease severity thresholds defined by Reynolds in the RADS-2 professional manual) (Cohen's *d* = 0.5) which is conservative compared to Cohen's *d* = 0.83 in our pilot study (27) with an SD of 15.5, 107 subjects are needed. Assuming a dropout rate of 20%, based on 13% in our clinical pilot study (27), 134 participants will be recruited (*N* = 67 in each study arm). The study will not be powered to compare age-groups.

## Discussion

This study will be the first RCT to examine the clinical effectiveness of a TARA compared to standard treatment for adolescents and young adults with depressive disorders. Recruitment and treatment will start in the spring of 2021 and the last follow-up that will be presented in the primary manuscript is expected to be completed in 2025. The present article presents the analyses that will be published in the primary manuscript. The statistical analysis plan was written to prepare for future analyses and to increase transparency of scientific conduct, thereby allowing others to comment on our proposed strategy.

Depression in adolescents and young adults is prevalent and highly debilitating, while treatment options are still far from satisfactory. TARA may be of value for adolescents and young adults with depression given the neuroscientific base and neurodevelopmental adaptions, the focus on autonomic and emotional self-regulation including in interpersonal relationships, the contextual base in socio-ecological theory as well as the value based behavioral activation approach (25).

Our study has some limitations. First, our primary outcome measure will be self-reported, which in light of limited masking makes it subject to bias. Given that both study arms include complex interventions, to choose a more objective outcome measure, such as assessment by an independent clinician as our primary outcome, could have been preferable. We argue that self-report gives more information compared to clinician ratings or parental report for internalizing disorders in this age-group (97, 98) and include clinician rating as a secondary outcome measure. Also, psychometric validation of all measures is generally required for the entire study population. Even though the inclusion of participants in the age range of 15–22 years of age will be a major contribution to this field, it does make it difficult to find outcome measures that are validated for all participants. We are currently collecting data on a number of self-report instruments in concurrent studies and plan to publish validity-data on the primary outcome and several other of the included self-report questionnaires for the full age-range of this study.

Second, the major incentive for a patient to participate, apart from altruism and the monetary reimbursements, may be to receive the novel intervention TARA. If participants are allocated to standard treatment, they may be more likely to withdraw from the trial (causing attrition bias) or exhibit disappointment bias when reporting outcomes. An attrition analysis will be conducted to assess reasons for attrition, to explore group-differences in attrition and explore differences between completers and non-completers on baseline measures. Furthermore, it is made very clear before inclusion that it is unknown what treatment is more effective, which should counteract the described tendencies.

Third, although we have an active control group, waiting times to treatment and the length and intensity of the treatment given may come to differ between the two trial arms. It is possible that some participants allocated to standard treatment will, in reality, become wait listed controls. This may artificially strengthen the results in favor of TARA. The content of standard treatment may also change over time, both within and between participants, making it difficult to describe as well as potentially impacting the outcome measures. The advantage of our approach is that we compare TARA to the treatment that is actually provided at CAP and YC clinics today, and participants' medical records will be thoroughly analyzed to describe the content of the standard treatment arm. Increased therapeutic efforts and improved results of standard treatment could occur simply as a result of being observed, i.e., the Hawthorne effect (99). This should however not impact the results to the advantage of TARA. Significant contamination of study arms is not expected, even if some therapists may come to deliver treatment in both study arms.

Fourth, since the prescription rate of SSRIs has increased in the last decade and SSRIs are often initiated in primary care in Sweden (100), it will not be feasible to recruit only medication-naïve patients to this study. Given that some participants will not be willing to discontinue their medication and some may require hyperbolic tapering regimens (101), it is possible that TARA in some cases will become an add-on treatment to SSRIs. Our pragmatic approach increases external validity by making the results relevant to a larger population and for those who successfully discontinue medication that is a beneficial outcome in itself.

This study has some major strengths, including the RCT-design with an active control setting based on the currently recommended standard treatment. Also, young adults with MDD/PDD have not previously been considered for treatments using neuroscientific age-adaption. The age group targeted in this study is neurodevelopmentally distinct from adults and covers the critical age-range of 15–22, when some of the most sophisticated depression-related higher-order neurodevelopment occurs (23). Also, behavioral data and symptom severity ratings will be collected in multiple ways, including qualitatively as well as quantitatively with self-report and clinician-rating. Bio-indicators of depression will be measured in blood, plasma and hair, which together with physiological measures such as accelerometry, HRV and MRI will enable further elucidation of potential mechanisms of change. Mediation analyses may also help disentangle causal relationships with regard to active components of TARA. Measuring the amount of home practice in the TARA-arm will enable analysis of dose-response relationships. Furthermore, the 2-year follow-up will allow for an interpretation of the long-term effects of the different treatments. Moreover, our multi-center approach includes both specialized CAP-clinics and primary care youth clinics and engages therapists with different experiences, education, and background, which will increase generalizability. If this approach is shown to be effective, the online format of TARA will facilitate a large-scale future implementation. Finally, while this study has some aspects that are explanatory in nature, the main approach is pragmatic, which will hopefully make the results relevant to policymakers.

## Conclusion

Given the increasing impact that depressive disorders have on the global burden of disease, and the lack of efficacious treatment options for adolescents, rigorous efforts need to be directed toward the evaluation of novel treatments. TARA is an appealing candidate with both substantial theoretical and practical underpinnings. This study protocol and analysis plan were written to avoid the risk of selective outcome reporting bias and data-driven research. By submitting this manuscript before recruiting our first participant it is hoped that the results will be as robust and transparent as possible. The results of this trial may optimize the clinical treatment of depression and impact daily clinical practice to improve patient-outcomes. We argue that this study will extend the current knowledgebase regarding treatments for adolescents and young adults with depression.

## Ethics Statement

The studies involving human participants were reviewed and approved by The Regional Ethical Review Board, situated in Umeå, Dnr 2018-221-31M and 2019-000423, and the National Ethical Review Board, Dnr 2020-05734. Written informed consent to participate in this study is provided by the participants, where applicable, by legal guardian(s).

## Author Contributions

EE and EH performed the conception and design of the study and drafted the manuscript. GG, RS, and IB critically revised the study protocol and analysis plan. All authors approved the final manuscript.

## Funding

This study was funded by the County Council of the Region Västerbotten, the County Council of the Region Västernorrland, municipality of Örnsköldsvik and the Kempe foundation (LVNFOU933598), the Swedish Society of Medicine (SLS-935854), Lars Jacob Boëthius foundation, and the Oskar-foundation. The funders have no role in the design, methods, subject recruitment, data collections, analysis, or preparation of manuscripts in the project.

## Conflict of Interest

The authors declare that the research was conducted in the absence of any commercial or financial relationships that could be construed as a potential conflict of interest.

## Publisher's Note

All claims expressed in this article are solely those of the authors and do not necessarily represent those of their affiliated organizations, or those of the publisher, the editors and the reviewers. Any product that may be evaluated in this article, or claim that may be made by its manufacturer, is not guaranteed or endorsed by the publisher.

## Footnotes

^1^ https://www.mindmedia.com/en/products/nexus-10-mkii/

^2^ https://www.kubios.com/

^3^ https://www.camntech.com/products/motionwatch/motionwatch-8-overview

## Abbreviations

AEs: Adverse Events
CAP: Child and Adolescent Psychiatry
CONSORT: Consolidated Standards Of Reporting Trials
CDRS-R: Children's Depression Rating Scale-Revised
CRC: Clinical Research Center
DSM-IV: Diagnostic and Statistical Manual of Mental Disorders, 4th edition
DSM-5: Diagnostic and Statistical Manual of Mental Disorders, 5th edition
HRV: Heart Rate Variability
ICD-10: International Classification of Diseases 10th revision
MDD: Major Depressive Disorder
MRI: Magnetic Resonance Imaging
RADS-2: Reynolds Adolescent Depression rating Scale 2
RCT: Randomized Controlled Trial
RDoC: Research Domain Criteria
SAEs: Serious Adverse Events
SPIRIT: Standard Protocol Items: Recommendations for Interventional Trials
SSRIs: Selective Serotonin Reuptake Inhibitors
TARA: Training for Awareness Resilience and Action
YC: Youth Clinic.
